# Factors modifying the risk for developing acute skin toxicity after whole-breast intensity modulated radiotherapy

**DOI:** 10.1186/1471-2407-14-711

**Published:** 2014-09-25

**Authors:** Sofie De Langhe, Thomas Mulliez, Liv Veldeman, Vincent Remouchamps, Annick van Greveling, Monique Gilsoul, Eline De Schepper, Kim De Ruyck, Wilfried De Neve, Hubert Thierens

**Affiliations:** Department of Basic Medical Sciences, Ghent University, Proeftuinstraat 86, 9000 Ghent, Belgium; Department of Radiotherapy, Ghent University Hospital, De Pintelaan 185, 9000 Ghent, Belgium; Department of Radiotherapy, Clinique et Maternité St Elisabeth, Place Louise Godin, 15, 5000 Namur, Belgium

**Keywords:** Acute skin toxicity, Breast cancer, Genetic polymorphisms, Large breast size, Radiotherapy

## Abstract

**Background:**

After breast-conserving radiation therapy most patients experience acute skin toxicity to some degree. This may impair patients’ quality of life, cause pain and discomfort. In this study, we investigated treatment and patient-related factors, including genetic polymorphisms, that can modify the risk for severe radiation-induced skin toxicity in breast cancer patients.

**Methods:**

We studied 377 patients treated at Ghent University Hospital and at ST.-Elisabeth Clinic and Maternity in Namur, with adjuvant intensity modulated radiotherapy (IMRT) after breast-conserving surgery for breast cancer. Women were treated in a prone or supine position with normofractionated (25 × 2 Gy) or hypofractionated (15 × 2.67 Gy) IMRT alone or in combination with other adjuvant therapies. Patient- and treatment-related factors and genetic markers in regulatory regions of radioresponsive genes and in *LIG3*, *MLH1* and *XRCC3* genes were considered as variables. Acute dermatitis was scored using the CTCAEv3.0 scoring system. Desquamation was scored separately on a 3-point scale (0-none, 1-dry, 2-moist).

**Results:**

Two-hundred and twenty patients (58%) developed G2+ dermatitis whereas moist desquamation occurred in 56 patients (15%). Normofractionation (both p < 0.001), high body mass index (BMI) (p = 0.003 and p < 0.001), bra cup size ≥ D (p = 0.001 and p = 0.043) and concurrent hormone therapy (p = 0.001 and p = 0.037) were significantly associated with occurrence of acute dermatitis and moist desquamation, respectively. Additional factors associated with an increased risk of acute dermatitis were the genetic variation in *MLH1* rs1800734 (p=0.008), smoking during RT (p = 0.010) and supine IMRT (p = 0.004). Patients receiving trastuzumab showed decreased risk of acute dermatitis (p < 0.001).

**Conclusions:**

The normofractionation schedule, supine IMRT, concomitant hormone treatment and patient related factors (high BMI, large breast, smoking during treatment and the genetic variation in *MLH1* rs1800734) were associated with increased acute skin toxicity in patients receiving radiation therapy after breast-conserving surgery. Trastuzumab seemed to be protective.

## Background

Breast-conserving therapy with the adjuvant use of radiotherapy (RT) has gained an established role in the treatment for early-stage breast cancer with excellent long-term local control and survival [1]. During or shortly after the course of breast cancer RT, a large portion of the patients will experience acute radiation dermatitis to some degree, varying from mild to brisk erythema with or without moist desquamation and occasionally ulceration of the skin [2]. There is accumulating clinical evidence that acute reactions are associated with the development of late toxicity: Lilla et al. showed that telangiectasia are in fact late sequelae of moist desquamation and acute erythema is shown to be a risk factor for poor cosmetic outcome [3–5]. Though the skin is not a dose-limiting tissue, skin toxicity is associated with impairment of patients’ quality of life, causes pain and discomfort and limits activities [2, 6]. The challenge is to minimize these side effects without losing efficacy of the treatment.

Over the years, many attempts have been made to reduce the number of patients experiencing acute skin toxicity and inferior cosmetic outcome by introducing improved radiation techniques, such as intensity-modulated radiotherapy (IMRT). This technique has been shown to be superior over conventional wedge-based whole breast irradiation by delivering a more homogenous dose through the breast and removing the radiation hot spots; it results in an approximately 20% reduction of the frequency of moist desquamation [6, 7]. Large breast size significantly contributes to dose inhomogeneity, hot spots and toxicity [7, 8]. The variation in clinical response is, however, only partly explained by treatment factors such as radiation dose, fractionation scheme, and concomitant therapies. Patient-related features (e.g. bra cup size and body mass index (BMI)) also play a role together with an unknown contribution from genetic factors. Up to now there are no data available to estimate directly the heritability of clinical radiosensitivity based upon family history of radiotherapy toxicity, but it is likely to be somewhat lower than for chromosomal and cellular radiosensitivity, which have been calculated to be 58-78% [9].

Acute toxicity is initiated by depletion of acutely responding epithelial tissues and damage to microvessels [10]. Numerous studies have reported on genetic variations modifying the clinical radiosensitivity risk, predominantly in pathways based on mechanistic understanding of the radiation pathogenesis (reviewed in [11]). In the present study, single nucleotide polymorphisms (SNPs) in genes involved in major DNA repair pathways (*LIG3*, *XRCC3, MLH1*) and in regulatory regions that influence the expression levels of radioresponsive genes are considered [12–16].

To gain a better insight into the development of radiation-induced dermatitis and moist desquamation, we evaluated the association between patient and treatment features with these endpoints. The association between SNPs and the different clinical endpoints was also studied.

## Methods

The study population consists of 377 breast cancer patients treated with adjuvant IMRT with curative intent after breast-conserving surgery (stage T1-3, N0-1, M0). Of them, 282 breast cancer patients were treated at the Ghent University Hospital (GUH) and 95 patients were treated at ST.-Elisabeth Clinic and Maternity (CMSE) in Namur. Patients’ follow-up ranged from 1 month after the end of RT to 41 months after the end of RT (median = 18 months).

At GUH, patients were treated in prone or supine position using a multi-beam IMRT technique in supine position and a tangential 2-beam field-in-field IMRT technique in prone position as described previously [17]. The whole breast was treated with hypofractionated radiotherapy (40.05 Gy in 15 fractions [18]) with 6-MV photons of an Elekta Synergy linear accelerator (Crawley, United Kingdom). An additional photon boost of 10 Gy in 4 fractions to the tumour bed was given to 75% of the patients. For the prone patient setup, a unilateral breast holder (Van De Velde, Schellebelle, Belgium) and a prone breast board (Orfit Industries) were used [19]. Twenty-two patients were treated in prone position with voluntary moderate deep inspiration breath hold. At CMSE Namur, a sliding window tangential field-IMRT technique was used associated with moderate deep inspiration breath hold whenever the primary beam intersected the heart as previously described by Remouchamps et al. [20]. Patients with self-reported bra cup size ≥ D received normofractionated radiotherapy (50.00 Gy in 25 fractions), women with bra cup size < D received hypofractionation or normofractionation according to the preference of the radiation oncologist (n = 28). More than 90% received an additional boost of 10 Gy in 4 fractions with electron beams. Nodal irradiation was performed by a complex multi-beam IMRT or arc technique at GUH, at CMSE Namur, a one point setup with 4 beams with dynamic intensity modulation in the beams was used.

### Adjuvant systemic therapy

Adjuvant hormone therapy, consisting of tamoxifen or aromatase inhibitors, was administered in most patients concomitantly with IMRT. The others received hormone therapy sequentially after IMRT. Patients who received adjuvant chemotherapy, combination of antracyclines and taxanes, completed chemotherapy before IMRT, while trastuzumab was allowed concomitantly with IMRT.

### Data collection

Data on patients’ medical history, tumor and treatment characteristics were collected prospectively. Table 1 gives an overview of the patient characteristics for patients treated at GUH and CMSE Namur.

**Table 1:** **Patient characteristics for patients treated at GUH and CMSE Namur**

		GUH		CMSE Namur
		(n = 282)		(n = 95)
Age (years)				
	Median	57.5		59.0
	Range	30-82		35-82
Bra cup size				
	Small	A	13 (4.6)	3 (3.2)
		B	85 (30.2)	33 (34.7)
		C	101 (35.8)	34 (35.8)
	Large	D	53 (18.8)	16 (16.8)
		E	16 (5.7)	5 (5.3)
		F	7 (2.5)	3 (3.2)
		G + H	2 (0.6)	1 (1.0)
	*Missing*		*5*	*0*
BMI				
	Median	25.5		26
	Range	16-50		16-38
	*Missing*	2		0
Menstruation				
	No	235 (83.3)		76 (80.0)
	Yes	45 (16.0)		18 (18.9)
	*Missing*	*2*		*1*
Smoking during RT				
	No	244 (86.5)		79 (83.2)
	Yes	35 (12.4)		16 (16.8)
	*Missing*	*3*		*0*
Diabetes				
	No	254 (90.1)		84 (88.4)
	Yes	22 (7.8)		11 (11.6)
	*Missing*	*6*		*0*
Hypertension				
	No	196 (69.5)		66 (69.5)
	Yes	81 (28.7)		29 (30.5)
	*Missing*	*5*		*0*
Fractionation				
	Normo	0		45 (47.4)
	Hypo	282		50 (52.6)
	*Missing*	*0*		*0*
Treatment position				
	Supine	195 (69.1)		95 (100.0)
	Prone	87 (30.9)		0
	*Missing*	*0*		*0*
Boost				
	No	64 (22.7)		7 (7.4)
	Yes	218 (77.3)		88 (92.6)
	*Missing*	*0*		*0*
Nodal irradiation				
	No	241 (85.5)		87 (80.6)
	Yes	41 (14.5)		21 (19.4)
	*Missing*	*0*		*0*
Hormonal therapy				
	No	46 (16.3)		25 (26.3)
	Concomitant	236 (83.7)		7 (7.4)
	Sequential (after IMRT)	0		63 (66.3)
	*Missing*	*0*		*0*
Chemotherapy				
	No	188 (66.7)		55 (57.9)
	Yes	94 (33.3)		40 (42.1)
	*Missing*	*0*		*0*
Trastuzumab				
	No	257 (91.1)		83 (87.4)
	Yes	25 (8.9)		12 (12.6)
	*Missing*	*0*		*0*

*Abbreviations:* *GUH* Ghent University Hospital, *CMSE* ST.-Elisabeth Clinic and Maternity, *BMI* Body Mass Index.

Data are given as no. (%) unless otherwise indicated.

Acute toxicity was assessed weekly during treatment and at 1–2 weeks after treatment. The reported toxicity represents the maximal reported acute toxicity, either during or after completion of IMRT. Acute dermatitis was documented according to a standard protocol using the Common Terminology Criteria for Adverse Events (CTCAE) v3.0 scoring system. This grades patients with mild erythema or dry desquamation as 1, moderate to brisk erythema or patchy moist desquamation mostly confined to the skin folds as 2 and confluent moist desquamation as 3. Desquamation was scored separately on a 3-point scale (0-none, 1-dry, 2-moist). Grade 2–3 toxicity was considered clinically relevant and was included in the analysis. Genomic DNA was isolated from a fresh blood sample taken before start of radiotherapy, using the Puregene genomic DNA purification kit (Gentra Systems, Minneapolis, MN). The study was approved by the local ethics committees (Ghent University Hospital EC 2009/424, EC 2009/184) and all study patients provided written informed consent.

### Selection of candidate genes/polymorphisms and genotyping

Eight candidate polymorphisms were selected for genotyping (Table 2). Of these, five SNPs (rs3888929, rs4867592, rs7970524, rs12003093, rs4760658) were chosen as they putatively affect the expression levels of radiation-responsive genes directly, or by *trans* effects, based on genetic linkage and association analysis as described previously by Smirnov et al. The authors suggested that those regulatory variants might be able to contribute to the development of genetic tools for radiosensitivity [16]. The other SNPs were chosen based on their previous association with toxicity induced by radiotherapy or methylating agents (*XRCC3* rs861539, *LIG3* rs3744355, *MLH1* rs1800734) [12–15]. Genotyping was performed using restriction fragment length polymorphism analyses, high resolution melting curve analyses, single base extension techniques or direct sequencing. For reproducibility control, 15% of all samples were duplicated. The concordance rate between duplicate samples was 100%. Primers details are available on request. Tests for deviation from Hardy-Weinberg equilibrium, for the entire sample showed that the rs4867592 SNP had a p-value <0.0001 and was excluded from further analyses.

**Table 2:** **Characteristics of the SNPs**

Gene or gene regulator	rs number	MAF*	Nucleotide substitution	Genomic location	Amino acid substitution	Reference
*LIG3*	rs3744355	9.1	G > C	5′-flanking	-	[12, 13]
*MLH1*	rs1800734	22.6	G > A	5′-UTR	-	[14]
*XRCC3*	rs861539	39.0	C > T	Coding	Thr241Met	[15]
*PHLDA3*	rs3888929	30.3	G > A	Unknown	-	[16]
*LCP2*	rs4867592	19.1	C > A	Unknown	-	[16]
*LTHA4*	rs7970524	25.1	T > C	5′-flanking	-	[16]
*NDUFB6*	rs12003093	23.4	A > G	Unknown	-	[16]
*VDR*	rs4760658	36.6	A > G	Intronic	-	[16]

*Minor allele frequency in Caucasian population.

### Statistical analysis

The studied endpoints were development of acute radiation-induced dermatitis (CTCAE G2+) and moist desquamation. For the clinical association analysis, univariate analysis was initially carried out to assess the relationship between patient- (age, bra cup size (A + B + C vs. ≥D), BMI, menstruation, smoking during RT, diabetes, hypertension) and treatment-related factors (fractionation scheme, treatment position, boost dose to tumour bed, nodal irradiation, hormone therapy, chemotherapy and trastuzumab) and the endpoints. Patients with and without G2+ acute skin toxicity were compared by means of the Mann–Whitney test for continuous variables and the χ^2^-test for categorical variables. Power calculations were performed with Power for Genetic Association analyses [21]. For these we took into account: the incidence of dermatitis (58%) or moist desquamation (15%) observed in our cohort, the lowest minor allele frequency (9%) of the considered SNPs, a probability adjusted by the number of SNPs (α = 6.25 × 10^-3^) under a dominant genotypic test, and a genotype relative risk of ≥1.5. This resulted in a power of 94.3% for acute dermatitis and 60.9% for moist desquamation. To assess the independent effect of each polymorphism, unconditional logistic regression analyses were performed to calculate crude ORs. The Benjamini-Hochberg (BH) procedure was used to control for multiple testing (i.e. 43 tests per endpoint: 28 genetic and 15 clinical parameter tests) to reduce the risk of finding false-positive associations. Variables with p < 0.05 were tested in a multivariate logistic regression analysis. Statistical analyses were performed using SPSS 17.0 software (SPSS Inc., Chicago, IL). R library *multtest* ( http://www.r-project.org/) was used to perform the multiple testing analyses.

## Results

Acute radiation-induced skin toxicity data were available for all 377 patients. Two-hundred twenty patients (58%) developed G2+ dermatitis. The occurrence of dermatitis did not differ between both centres (GUH: 57% (162/282), CSME: 61% (58/95)). Moist desquamation (patchy or confluent) occurred in 56 patients (15%) and differed between both centres: 10% of the patients treated at GUH and 30% of the patients treated at CMSE (p < 0.001).

### Acute radiation-induced skin toxicity

Table 3 depicts the parameters associated with acute G2+ dermatitis, in univariate analysis. Bra cup size ≥ D (p < 0.001), BMI (p < 0.001) and smoking during RT (p = 0.029) were associated with the development of G2+ dermatitis. Irradiation of the nodal region (p = 0.006) and the use of concomitant hormone therapy (p = 0.041) were also associated with an increased risk of acute dermatitis, with no difference in incidence between aromatase-inhibitors and tamoxifen. In contrast, patients receiving trastuzumab or having received chemotherapy seem to be less prone to the development of RT-induced acute dermatitis (p = 0.003 and p = 0.045, respectively). Furthermore, patients treated with hypofractionated radiotherapy develop less dermatitis when compared to patients treated in the normofractionated regimen (p < 0.001). And, patients treated in prone position developed less dermatitis than patients treated supine (p = 0.002). In multivariate analysis, chemotherapy and nodal irradiation were no longer significant (Table 4).

**Table 3:** **Associations between patient- and therapy-related characteristics and acute G2+ dermatitis**

		All (n = 377)	G0-1 (n = 157)	G2+ (n = 220)	p-value	p _BH_-value
Bra cup size						
	A + B + C	269 (71.4)	130 (48.3)	139 (51.7)		
	≥D	103 (27.3)	26 (25.2)	77 (74.8)	**<0.001**	**0.001**
BMI						
	Median	26	24	26		
	Range	16-50	16-37	16-50	**<0.001**	**0.001**
Smoking during RT						
	No	323 (85.7)	141 (43.7)	182 (56.3)		
	Yes	51 (13.5)	14 (27.5)	37 (72.5)	**0.029**	0.156
Fractionation						
	Normo	45 (11.9)	6 (13.3)	39 (86.7)		
	Hypo	332 (88.1)	151 (45.5)	181 (54.5)	**<0.001**	**<0.001**
Treatment position						
	Supine	290 (76.9)	108 (37.2)	182 (62.8)		
	Prone	87 (23.1)	49 (56.3)	38 (43.7)	**0.002**	**0.019**
Nodal irradiation						
	No	315 (83.6)	141 (44.8)	174 (55.2)		
	Yes	62 (16.4)	16 (25.8)	46 (74.2)	**0.006**	**0.037**
Hormonal therapy						
	No	71 (18.8)	39 (54.9)	32 (45.1)		
	Concomitant	243 (64.5)	94 (38.7)	149 (61.3)		
	Sequential (after IMRT)	63 (16.7)	24 (38.1)	39 (61.9)	**0.041**	0.207
	*Hormones (concomitant)*					
	*Tamoxifen*	*155*	*62 (40.0)*	*93 (60.0)*		
	*Aromatase inhibitor*	*85*	*32 (37.6)*	*53 (62.4)*		
Chemotherapy						
	No	243 (64.5)	92 (37.9)	151 (62.1)		
	Yes	134 (35.5)	65 (48.5)	69 (51.5)	**0.045**	0.215
Trastuzumab						
	No	340 (90.2)	133 (39.1)	207 (60.9)		
	Yes	37 (9.8)	24 (64.9)	13 (35.1)	**0.003**	**0.026**

*Abbreviations:* *G* CTCAEv.3 grade, *BMI* Body Mass Index; p_BH_ = corrected p-value by Benjamini-Hochberg procedure.

Data are given as no. (%) unless otherwise indicated. P<0.05 is considered significant and is showed in bold.

**Table 4:** **Multivariate analysis for G2+ dermatitis and moist desquamation**

Clinical/genetic factor	Acute G2+ dermatitis		Moist desquamation	
	OR	p-value	OR	p-value
**Center** (CMSE vs. GUH)	-	-	3.206	0.158
**BMI**	**1.088**	**0.003**	**1.170**	**<0.001**
**Bra cup size** (cup ≥ D vs. cup A + B + C)	**2.833**	**0.001**	**2.146**	**0.043**
**Smoking** (yes vs. no)	**2.711**	**0.010**	**-**	**-**
**Fractionation** (hypo vs. normo)	**0.083**	**<0.001**	**0.096**	**<0.001**
**Treatment position** (prone vs. supine)	**0.399**	**0.004**	0.373	0.074
**Hormone therapy**				
No	1		1	
Concomitant	**3.207**	**0.001**	**4.770**	**0.037**
Sequential (after IMRT)	1.003	0.994	1.078	0.901
**Nodal irradiation** (yes vs. no)	1.975	0.100	-	-
**Chemotherapy** (yes vs. no)	0.954	0.877	-	-
**Trastuzumab** (yes vs. no)	**0.177**	**<0.001**	-	-
***MLH1*** **rs1800734 G > A**				
GG	1		-	
GA	**0.492**	**0.008**	-	-
AA	0.537	0.232	-	-

*Abbreviations:* *GUH* Ghent University Hospital, *CMSE* Clinic Maternity Sainte-Elisabeth, *BMI* Body Mass Index, *MLH1* MutL protein homolog 1.

P<0.05 is considered significant and is showed in bold.

For moist desquamation, univariate significant associations were found with bra cup size ≥ D (p < 0.001), BMI (p < 0.001), normofractionation (p < 0.001), supine positioning (p = 0.002), concurrent hormone therapy (p = 0.004) and CSME center (p < 0.001) (Table 5). In multivariate analysis (Table 6), bra cup size ≥ D, BMI, fractionation and hormone therapy remained statistically significant. Treatment center was no longer significantly associated with moist desquamation due to the fact that the normofractionated schedule was only prescribed at CMSE.

**Table 5:** **Associations between patient- and therapy-related characteristics and moist desquamation**

		All patients				
		All (n = 377)	No (n = 321)	Yes (n = 56)	p-value	p _BH_-value
Bra cup size						
	A + B + C	269 (71.4)	242 (90.0)	27 (10.0)		
	≥D	103 (27.3)	76 (73.8)	27 (26.2)	**<0.001**	**0.001**
BMI						
	Median	26	25	29		
	Range	16-50	16-40	21-50	**<0.001**	**<0.001**
Fractionation						
	Normo	45 (11.9)	22 (48.9)	23 (51.1)		
	Hypo	332 (88.1)	299 (90.1)	33 (9.9)	**<0.001**	**<0.001**
Treatment position						
	Supine	290 (76.9)	239 (82.4)	51 (17.6)		
	Prone	87 (23.1)	82 (94.3)	5 (5.7)	**0.002**	**0.019**
Hormonal therapy						
	No	71 (18.8)	62 (87.3)	9 (12.7)		
	Concomitant	243 (64.5)	214 (88.1)	29 (11.9)		
	Sequential (after IMRT)	63 (16.7)	45 (71.4)	18 (28.6)	**0.004**	**0.029**
	*Hormones (concomitant)*					
	*Tamoxifen*	*155*	*139 (89.7)*	*16 (10.3)*		
	*Aromatase inhibitor*	*85*	*74 (87.1)*	*11 (12.9)*		

*Abbreviations:* *GUH* Ghent University Hospital, *CMSE* Clinic Maternity Sainte-Elisabeth, *BMI* Body Mass Index; p_BH_ = corrected p-value by Benjamini-Hochberg procedure.

Data are given as no. (%) unless otherwise indicated. P<0.05 is considered significant and is showed in bold.

**Table 6:** **Effect of** ***MLH1*** **rs1800734 on radiotherapy acute skin reactions**

		Acute G2+ dermatitis					Moist desquamation				
		G0-1 (n = 157)	G2+ (n = 220)	OR	p-value	p _BH_-value	No (n = 321)	Yes (n = 95)	OR	p-value	p _BH_-value
***MLH1*** **rs1800734**											
G > A	GG	81 (51.6)	146 (66.4)				189 (58.9)	38 (67.9)			
	GA	64 (40.8)	60 (27.3)	0.52	**0.004**	**0.029**	110 (34.3)	14 (25.0)	0.63	0.172	0.477
	AA	9 (5.7)	12 (5.5)	0.74	0.514	0.804	17 (5.3)	4 (7.1)	1.17	0.788	0.915
	*Missing*	*3 (1.9)*	*2 (0.9)*				*5 (1.6)*	*0*			
GG vs. GA + AA (dominant)				0.55	**0.005**	**0.033**			0.71	0.257	0.575
GG + GA vs. AA (recessive)				0.94	0.889	0.936			1.35	0.600	0.860

*Abbreviations: MLH1* MutL protein homolog 1, p_BH_ = corrected p-value by Benjamini-Hochberg procedure.

Data are given as no. (%) unless otherwise indicated. P<0.05 is considered significant and is showed in bold.

### Genetic analysis

The only significant p-value, in univariate analysis, was for acute radiation-induced dermatitis with the GA genotype of rs1800734 in the *MLH1* gene with a BH-adjusted p-value of 0.029 (Table 6). Adjusting for above mentioned factors by multivariate regression analysis had no effect on the statistically significant association. None of the other SNPs had any effect on the risk of acute skin toxicity.

## Discussion

This study was performed to analyze the influence of treatment and patient-related factors on the development of acute radiation-induced skin toxicity. Bra cup size, BMI, smoking, treatment position, choice of RT schedule and the administration of adjuvant therapies seem to contribute to the variability in radiation skin toxicity. Also, the *MLH1* rs1800734 SNP was found to be significantly associated with the development of acute dermatitis.

Our data support the hypothesis that acute toxicity does not increase with moderate hypofractionation [22]. In fact, the occurrence of acute skin toxicity was significantly higher among patients treated with normofractionation compared to the hypofractionated schedule. There are only few reports studying hypofractionation in overweighed or large-breasted patients [23, 24]. We observe a 20% decrease in dermatitis and an even larger decrease (70%) in moist desquamation in large-breasted patients treated in supine position with hypofractionation compared to normofractionation (data not shown). Bra cup size and BMI were also confirmed as significant risk factors for the development of acute skin toxicity, in accordance with the majority of published reports [7, 8, 25–27]. Both are measures of breast volume as BMI was previously found to be strongly correlated with breast volume [27]. The association between larger breast volume and toxicity is thought to be due to dose inhomogeneity, high dose regions, and the bolus effect in the inframammary and axillary regions [8]. Due to the unavailability of dose homogeneity and hot spot data for the complete dataset, we were unable to test this for the total patient population, but the hypothesis is confirmed in a subset of the population [19]. Goldsmith et al. show that dose inhomogeneity is insufficient to explain the association and other factors like the presence of more adipose tissue might also play a role [25]. In prone position, the skin creases disappear, dose homogeneity is improved and hot spots are reduced leading to a reduction in acute skin toxicity [17]. In this study, we found a decrease in radiodermatitis and moist desquamation in patients treated with prone-IMRT. Especially patients with large breast sizes are expected to have a great benefit from prone-IMRT as shown by Mulliez et al. [19].

In this study, two types of adjuvant hormone therapy, tamoxifen or aromatase-inhibitors, were concurrently administered with radiotherapy to hormone receptor positive breast cancer patients. Present data show that use of hormone therapy is, regardless the type, associated with an increase in radiation-induced dermatitis. This is in accordance with a previous study investigating the effect of tamoxifen on acute skin reactions [26]. But in contrary with the COHORT randomized trial, that shows no difference between concurrent and sequential administration of letrozole; the latter was administered 3 weeks after RT when it is supposed that the radiosensitising effect of endocrine therapy is minimal [28]. Concurrent administration of trastuzumab and IMRT was found to be associated with lower rates of acute dermatitis in the present study. This finding needs to be put in perspective as it is in contradiction with the observation of a large randomized study that could not find a difference in acute toxicity [29]. Longer follow-up will be necessary to observe the effect of concurrent administration on cardiac toxicity.

Our study shows an association between the *MLH1* rs1800734 SNP and lower rates of acute radiation-induced dermatitis: heterozygotes are less present in the G2+ dermatitis group. The SNP maps 93 base pairs upstream of the *MLH1* transcription site in the core promoter*,* a region essential for maximum transcriptional activity [30]. The SNP was previously shown to be associated with acute myeloid leukemia after methylating chemotherapy for Hodgkin disease [15]. *MLH1* gene encodes MutL protein homolog 1 which is involved in DNA mismatch repair. Suga et al. found statistically significant associations with rs3744355 in the 5′ flanking region of the *LIG3* gene and acute radiation-induced skin reactions in the Japanese population and Murray et al. provided replicated evidence for this association in a European Caucasian population [12, 13]. We, however, could not confirm this association. Smirnov et al. hypothesized that regulatory variants might be able to contribute to the development of genetic tools to predict for radiosensitivity [16]. This could not be demonstrated in our study population.

Radiation-induced dermatitis includes erythema, edema, dry and moist desquamation as symptoms of inflammation probably triggered by cell death [31]. One of the shortcomings in this study is the fact that erythema was not measured objectively with a colorimeter. As the CTCAE criteria are based on subjective scoring, the difference between mild, moderate and brisk erythema is observer-dependent. This probably explains the large number of patients developing G2+ acute dermatitis when compared to other reports. A strength of our investigation is the nearly complete data set for a relatively large number of patients enrolled. Furthermore, patient recruitment as well as clinical outcome data collection were carried out prospectively. Although the associations hold after correcting for multiple testing, the results of this study should be validated in an independent study.

## Conclusion

A number of treatment and patient related factors are identified that modify the risk for the development of acute skin toxicity after whole-breast IMRT. Large bra cup, BMI, normofractionation and concomitant hormone therapy contribute to the development of moist desquamation. Patient related factors (high BMI, large breast, smoking during treatment and the genetic variation *MLH1* rs1800734), choice of RT schedule and the administration of adjuvant therapies affect the development of radiodermatitis.

## Abbreviations

BH: Benjamini-hochberg
BMI: Body mass index
CMSE: ST.-Elisabeth Clinic and Maternity
CTCAE: Common terminology criteria for adverse events
GUH: Ghent University Hospital
IMRT: Intensity-modulated radiotherapy
RT: Radiotherapy
SNP: Single nucleotide polymorphism.
